# Supplementary material for: Functional and Genetic Diversity of Bacteria Associated with the Surfaces of Agronomic Plants
Source: Plants (Basel). 2019 Apr 4;8(4):91. doi: 10.3390/plants8040091 (PMC6524364; doi:10.3390/plants8040091)
Supplement: Supplementary file 1 [file plants-08-00091-s001.zip › supplementary file-revised/Supplementary material.pdf]

Supplementary material

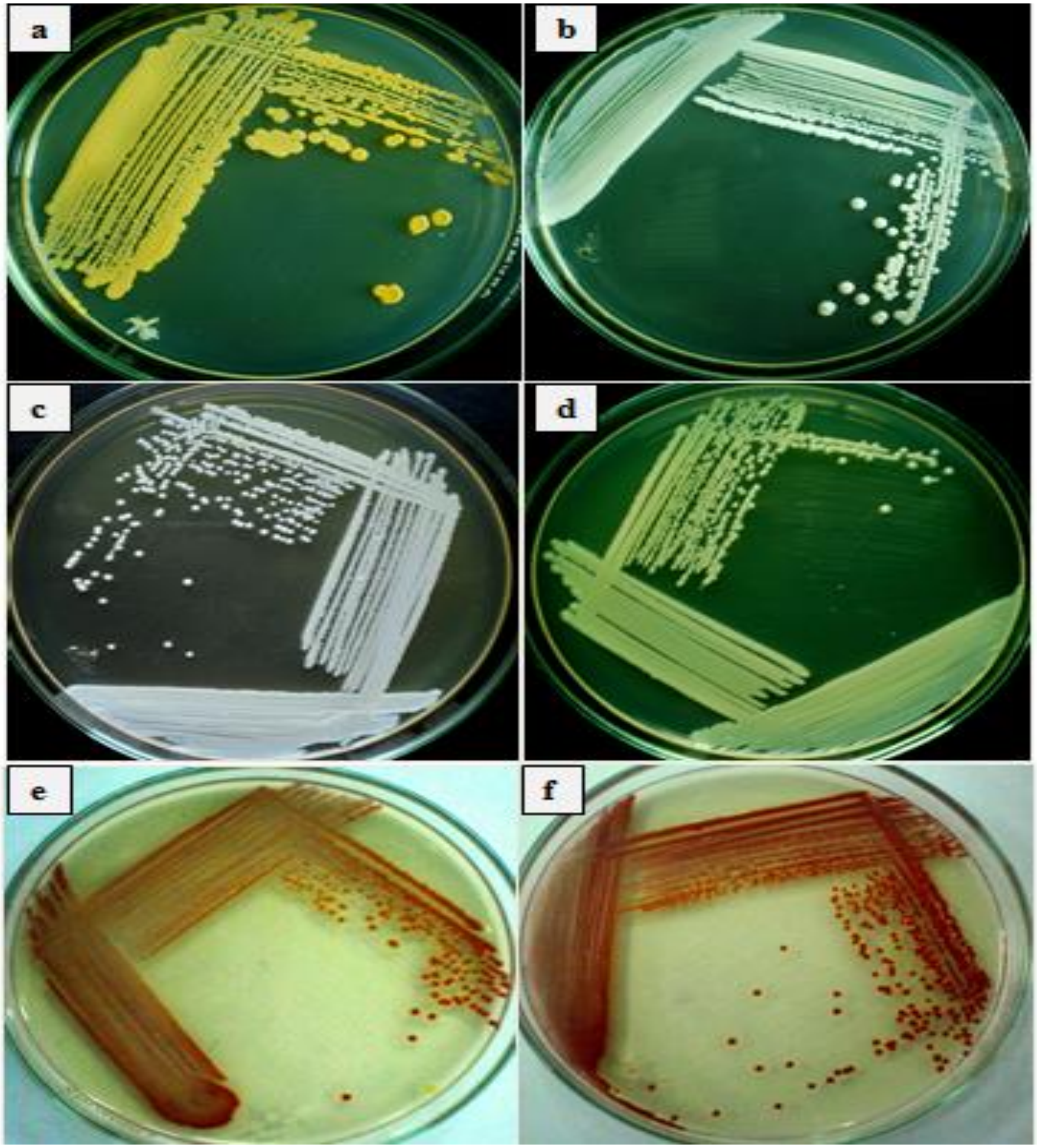

Figure S1. Purified bacterial colonies on LB agar. (a) = BPb-3, (b) = Lc-92, (c) = MCb-3, (d) = Mc-4, (e) = Lc-32, (f) = LCr-22

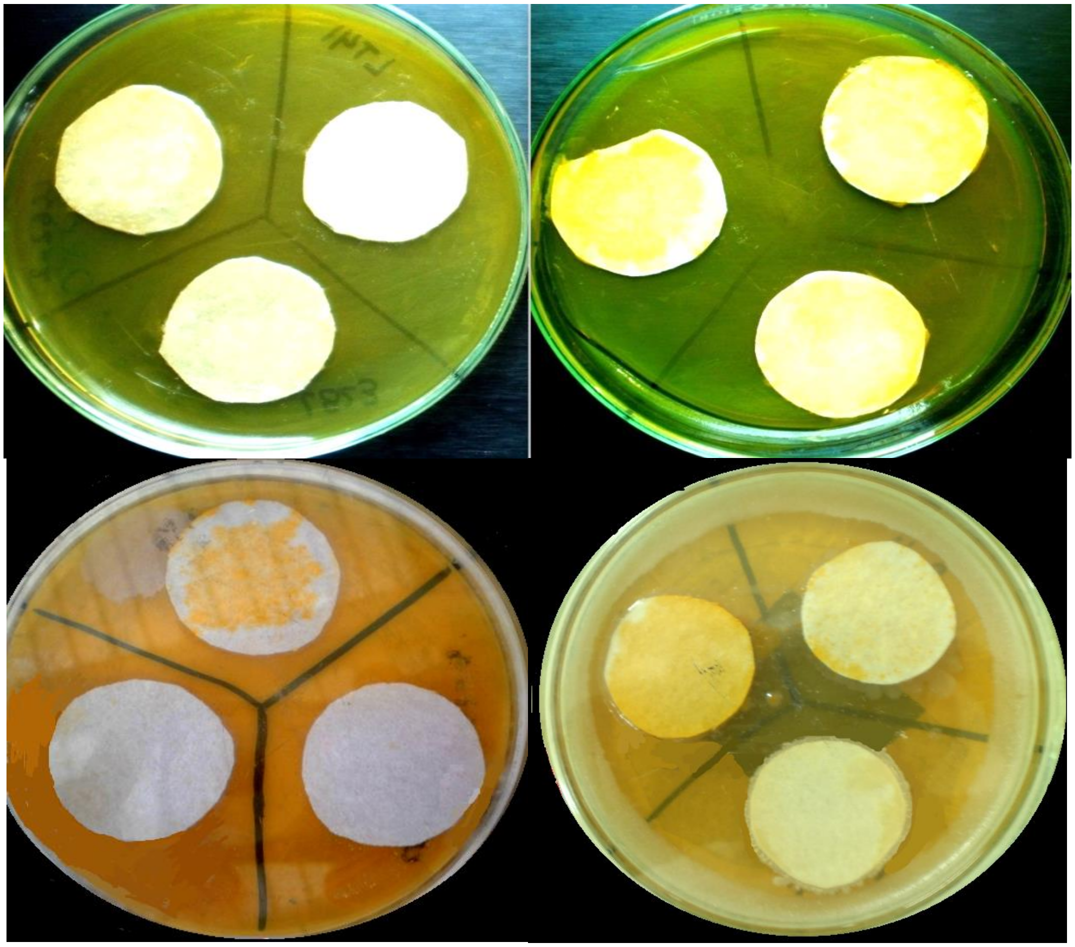

Figure S2. HCN production by different purified bacterial isolates that included BPb-5, BPc-1, Lt-73, Eb-8, MCb-3, MSt-8, BPc-3, Eb-9, Xt-3, Lb-61, Eb-10, Xc-5.

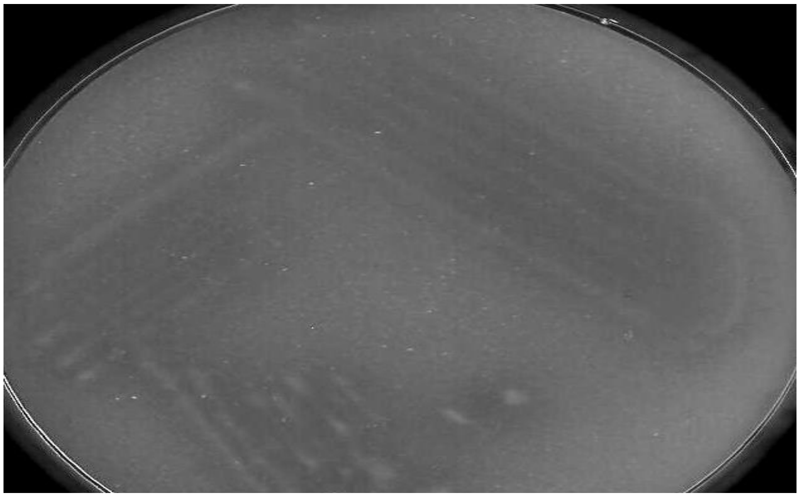

Figure S3. Phosphate solubilzation by *A. caloaceticus* Eb-8 on Pikovskaya medium.

Table S1. Auxin production and biofilm formation by plant associated bacteria

Serial No.	Strains	Auxin production with tryptophan				Biofilm production λ = 570	
		0 µg/ml		500 µg/ml			
1	Control	-		-		0.14	a
2	*Acinetobacter calcoaceticus* BPb-3	21.88	g-l	28.67	a-d	0.87	o-s
3	*Staphylococcus aureus* BPb-5	17.95	a-i	29.24	a-d	0.65	f-o
4	*Serratia rubidaea* BPc-1	20.60	d-l	27.24	a-d	0.33	abc
5	*Pantoea dispersa* BPc-3	16.52	a-i	21.17	a-d	0.50	b-i
6	*Bacillus cereus* BPc-4	13.46	abc	22.24	a-d	0.83	m-s
7	*S. equorum* BPt-5	16.58	a-i	20.24	a-d	0.30	ab
8	*Klebsiella pneumoniae* Eb-1	33.88	o	75.45	f	1.11	tu
9	*B. thuringiensis* Eb-10	15.18	a-f	21.00	a-d	0.38	b-e
10	*Pantoea vagans* Eb-2	18.62	a-i	42.45	b-e	0.47	b-h
11	*A. calcoaceticus* Eb-4	13.95	a-e	35.38	a-e	0.81	l-s
12	*Burkholderia cepacia* Eb-6	25.74	j-m	40.95	a-e	0.98	rst
13	*A. calcoaceticus* Eb-8	26.25	k-m	43.81	de	1.33	v
14	*B. cereus* Eb-9	16.36	a-i	28.95	a-d	0.58	d-l
15	*S. rubidaea* EMc-2	12.24	a	38.10	a-e	0.84	n-s
16	*B. anthracis* EMc-3	13.95	a-e	18.72	ab	0.35	a-d
17	*A. calcoaceticus* EMc-4	15.53	a-g	19.74	abc	0.78	k-q
18	*A. bouvetii* EMt-1	15.92	a-h	25.17	a-d	0.47	b-h
19	*Enterobacter amnigenus* EMt-5	16.81	a-i	24.24	a-d	0.39	b-e
20	*Arthrobacter nicotianae* Lb-41	22.88	i-l	27.67	a-d	3.42	y
21	*B. subtilis* Lb-61	19.53	b-j	35.24	a-e	0.55	c-k
22	*A. calcoaceticus* Lc-52	16.35	a-i	29.02	a-d	0.39	b-e
23	*S. rubidaea* Lcr-22	32.71	n-o	36.81	a-e	1.28	uv
24	*B. cereus* Lcw-22	18.25	a-i	40.24	a-e	1.48	v
25	*S. xylosus* Lt-41	27.47	l-n	33.53	a-d	1.43	v
26	*S. warneri* Lt-73	19.76	c-k	41.45	a-e	0.69	g-p
27	*S. rubidaea* Mc-2	30.12	m-o	77.81	f	1.03	st
28	*Stenotrophomonas maltophilia* Mc-3	18.81	a-i	29.02	a-d	1.02	st
29	*A. calcoaceticus* Mc-4	21.82	g-l	30.38	a-d	0.75	j-q

30	*S. arlettae* MCb-3	14.87	a-f	24.74	a-d	2.16	x
31	*Exiguobacterium mexicanum* MCb-4	20.80	e-k	22.95	a-d	2.21	x
32	*B. cereus* MCb-6	21.39	f-l	19.81	abc	0.61	e-n
33	*B. subtilis* MCb-8	17.67	a-i	40.96	a-e	0.71	h-p
34	*S. maltophilia* MCt-1	50.78	p	101.1	g	0.53	b-j
35	*Kluyvera cryocresens* MCt-5	13.68	a-d	58.17	ef	1.93	w
36	*S. ureilytica* MCt-6	18.76	a-i	33.60	a-d	0.39	b-e
37	*B. cereus* MSb-3	18.82	a-i	68.17	f	0.70	h-p
38	*B. anthracis* MSb-4	13.90	a-e	19.67	abc	1.29	uv
39	*Pantoea sp.* MSc-1	12.72	ab	17. 67	a	0.47	b-h
40	*S. warneri* MSc-5	18.87	a-i	28.46	a-d	1.40	v
41	*S. xylosus* MSt-1	17.78	a-i	30.95	a-d	0.73	i-q
42	*S. gallinarum* MSt-3	17.15	a-i	28.10	a-d	0.67	f-o
43	*A. calcoaceticus* MSt-6	15.45	a-g	19.38	abc	0.44	b-f
44	*B. cereus* MSt-7	21.17	f-l	42.36	b-e	0.46	b-g
45	*B. cereus* MSt-8	14.56	a-f	28.60	a-d	0.95	q-t
46	*B. cereus* PCt-1	22.64	h-l	97.24	g	0.59	d-m
47	*E. cloacae* PCt-2	34.28	o	71.10	f	0.91	p-t
48	*S. rubidaea* Xb-3	18.1	a-i	27.8	a-d	0.69	g-p
49	*B. anthracis* Xb-6	16.05	a-i	29.24	a-d	0.30	ab
50	*E. cloacae* Xc-3	31.77	m-o	43.35	cde	0.70	h-p
51	*Pseudomonas putida* Xc-5	16.52	a-i	20.93	a-d	0.47	b-h
52	*Citrobacter freundii* Xc-6	14.83	a-f	42.45	b-e	0.40	b-e
53	*Lysinibacillus. fusiformis* Xc-7	14.1	a-e	20.52	a-d	0.54	b-j
54	*S. xylosus* Xt-1	18.23	a-i	42.6	be	2.16	x
55	*C. werkmanii* Xt-3	20.53	a-i	29.3	a-d	1.22	u
56	*L. fusiformis* Xt-6	36.48	o	65.10	f	0.50	b-i

Means of three replicates. Different letters within the same column indicate significant difference between treatments using Duncan's multiple range test ($P \leq 0.05$).

Table S2. Phosphate solubilization and HCN production by different bacterial isolates

S. No	Strains	Phosphate solubilization	HCN production
1	*Acinetobacter calcoaceticus* BPb-3	++	+
2	*Staphylococcus aureus* BPb-5	-	-
3	*Serratia rubidaea* BPc-1	+	-
4	*Pantoea dispersa* BPc-3	-	+++
5	*Bacillus cereus* BPc-4	-	-
6	*S. equorum* BPt-5	-	++
7	*Klebsiella pneumoniae* Eb-1	++	-
8	*B. thuringiensis* Eb-10	-	+
9	*Pantoea vagans* Eb-2	+	-
10	*A. calcoaceticus* Eb-4	++++	+
11	*Burkholderia cepacia* Eb-6	+	-
12	*A. calcoaceticus* Eb-8	++++	++
13	*B. cereus* Eb-9	+	-
14	*S. rubidaea* EMc-2	+	-
15	*B. anthracis* EMc-3	-	+
16	*A. calcoaceticus* EMc-4	+	-
17	*A. bouvetii* EMt-1	-	+
18	*Enterobacter amnigenus* EMt-5	-	-
19	*Arthrobacter nicotianae* Lb-41	-	-
20	*B. subtilis* Lb-61	-	+++
21	*A. calcoaceticus* Lc-52	-	-
22	*S. rubidaea* Lcr-22	-	+
23	*B. cereus* Lcw-22	-	+
24	*S. xylosus* Lt-41	+	+
25	*S. warneri* Lt-73	-	-
26	*S. rubidaea* Mc-2	-	+
27	*Stenotrophomonas maltophilia* Mc-3	-	+
28	*A. calcoaceticus* Mc-4	-	-
29	*S. arlettae* MCb-3	-	++
30	*Exiguobacterium mexicanum* MCb-4	-	+
31	*B. cereus* MCb-6	-	+
32	*B. subtilis* MCb-8	++	-
33	*S. maltophilia* MCt-1	-	-
34	*Kluyvera cryocresens* MCt-5	-	-
35			

36	*B. cereus* MSb-3	++	-
37	*B. anthracis* MSb-4	+	-
38	*Pantoea sp.* MSc-1	+	+
39	*S. warneri* MSc-5	+	-
40	*S. xylosus* MSt-1	-	+
41	*S. gallinarum* MSt-3	-	-
42	*A. calcoaceticus* MSt-6	-	-
43	*B. cereus* MSt-7	-	-
44	*B. cereus* MSt-8	+	++
45	*B. cereus* PCt-1	-	+
46	*E. cloacae* PCt-2	-	+
47	*S. rubidaea* Xb-3	-	+
48	*B. anthracis* Xb-6	-	+
49	*E. cloacae* Xc-3	-	+
50	*Pseudomonas putida* Xc-5	-	-
51	*Citrobacter freundii* Xc-6	-	-
52	*Lysinibacillus. fusiformis* Xc-7	-	+
53	*S. xylosus* Xt-1	-	+
54	*C. werkmanii* Xt-3	-	-
55	*L. fusiformis* Xt-6	-	++

Number of '+' shows the intensity of the positive reaction where '++++' and '+' show the strongly and weakly positive reactions, respectively. The sign '-' shows the negative result.
